# Markers of increased cardiovascular risk in patients with chronic kidney disease

**DOI:** 10.1186/1476-511X-13-135

**Published:** 2014-08-21

**Authors:** Anna Gluba-Brzózka, Marta Michalska-Kasiczak, Beata Franczyk-Skóra, Marek Nocuń, Maciej Banach, Jacek Rysz

**Affiliations:** Department of Nephrology, Hypertension and Family Medicine, WAM University Hospital of Lodz, Medical University of Lodz, Zeromskiego 113, 90-549 Lodz, Poland; Department of Hypertension, Medical University of Lodz, Lodz, Poland; Nofer Institute of Occupational Medicine, Lodz, Poland

**Keywords:** Atherosclerosis, Chronic kidney disease, Calcification, CAD risk

## Abstract

**Background:**

Epidemiological studies have shown that chronic kidney disease (CKD) is an important risk factor for atherosclerosis and cardiovascular disease (CAD). The aim of the study was to determine markers of increased risk of CAD and to achieve a better understanding of agents implicated in the process of atherosclerosis in CKD patients.

**Methods:**

The study group consisted of a total of 139 patients with CKD while the control group comprised 45 healthy volunteers. Concentrations of osteoprotegerin, osteopontin, osteocalcin, matrix γ-carboxyglutamic acid (Gla) protein (MGP), fetuin A, matrix metalloproteinase-2 (MMP-2), matrix metalloproteinase-9 (MMP-9), tissue inhibitor of metalloproteinase-1 (TIMP-1), tissue inhibitor of metalloproteinase-2 (TIMP-2), ATP binding cassette transporter A1 (ABCA1), ATP binding cassette transporter G1 (ABCG1) and renalase were measured by the ELISA method.

**Results:**

We observed decreased levels of fetuin A (control *vs.* CKD group: 37.5 *vs.* 33.2 ng/ml, *p* = 0.018), and increased concentrations of osteocalcin (control *vs*. CKD group: 9.1 ± 6.0 vs. 13.6 ± 10.3 ng/ml, *p* = 0.05), MMP-2 (113.1 ± 75.0 *vs.* 166.0 ± 129.9 ng/ml, *p* = 0.045), TIMP-2 (22.1 ± 5.1 *vs.* 25.4 ± 7,0 ng/ml, *p* = 0.005) and renalase (251.0 ± 157 *vs*. 316.1 ± 155.3 ng/ml, *p* = 0.026). In patients with CKD (in comparison to control group), left ventricle ejection fraction: 53.0 ± 3,5% *vs.* 48.5%, p = 0.012) and calcification of the aortic valve (9.5% *vs.* 39.8%, *p* = 0.008) were observed more frequently.

**Conclusions:**

Decreased levels of fetuin A and increased concentration of osteocalcin, renalase, MMP-2 and TIMP-2 suggest that these factors may be involved in the pathogenesis of CAD in patients with CKD. Significantly increased indices of cardiac hypertrophy and its dysfunction in patients with CKD are indicators of pathological mechanisms occurring in cardiovascular system in this group of patients.

## Background

Chronic kidney disease (CKD) is a significant clinical problem worldwide, with constantly increasing incidence. Epidemiological studies have shown that chronic kidney disease is an important risk factor for cardiovascular disease [1]. Increased coronary artery disease (CAD) risk is associated with increased atherosclerosis, which is more frequent in patients with CKD, especially the dialysis ones, than in the general population. Cardiovascular disease is also the most common cause of mortality in this group of patients [2–4]. According to Foley et al. [3], the cardiovascular mortality rate in end stage renal disease (ESRD) patients is 10–20 times higher than in the general population after stratification for age, race and gender. At the time of starting renal replacement therapy, the prevalence of CAD is even higher [5–7]. The Hemodialysis (HEMO) Study revealed that ischaemic heart diseases (20.4%) as well as cardiac rhythm disorder (10.4%) were the most common cause of death in dialysed patient [8]. Increased risk of atherosclerosis in these patients is due to the overlapping of traditional risk factors of atherosclerosis with CKD-specific factors (i.e. altered calcium and phosphorus homeostasis, malnutrition, anaemia, hyperhomocysteinaemia, low serum albumin and high fibrinogen levels, as well as oxidative stress and inflammatory state) that further exacerbate atherosclerosis in the course of chronic kidney disease [9, 10]. So far, plaque formation mechanisms in this group of patients are not known, because patients with CKD were often excluded from the study. It seems that greater severity of atherosclerosis in patients with CKD may be associated with impaired lipid and calcium phosphate metabolism. Moreover, arterial calcification, which is an independent risk factor for acute coronary events, is observed even in young patients with CKD. Numerous studies have shown that bone metabolism plays an important role in patients with chronic kidney disease. Osteopontin, osteoprotegerin, fetuin A and matrix γ-carboxyglutamic acid protein (MGP) are the most important factors in the inhibition of calcifications. Additionally, matrix metalloproteinases (MMPs), which are involved in tissue remodelling, are implicated in the pathogenesis of both cardiovascular disease and kidney disease. Studies have shown that MMP-2, MMP-9 and tissue inhibitors of metalloproteinase -1 (TIMP-1) and -2 (TIMP-2) also play an important role in the pathogenesis of renal damage [11, 12].

The aim of the study was to determine markers of increased risk of CAD occurring in CKD patients and to achieve a better understanding of agents implicated in plaque formation in this group of patients. The study may support the identification of individuals who are at high risk of cardiovascular events in order to implement early prevention.

## Methods

The study group consisted of a total of 139 patients with CKD (30 patients with stage I/II CKD, 30 stage III CKD, 30 stage IV CKD and 49 stage V/dialysis) hospitalized in the Department of Nephrology, Hypertension and Family Medicine. The control group consisted of 45 healthy volunteers recruited from patients hospitalized due to causes other than CKD, CAD, hypertension, or diabetes mellitus. All subjects enrolled in this study were informed about its purpose and methodology and signed an informed consent form. The study was approved by the Bioethics Committee of the Medical University of Lodz (no. RNN/79/12/KB). The levels of studied proteins and biochemical markers were analysed in blood of all people involved in the study. The study excluded patients with diagnosed cancer, autoimmune diseases and those with a history of myocardial infarction. In the present study, concentrations of proteins involved in the processes of vessel wall calcification and bone metabolism disorders (osteoprotegerin [TECOMedical, no. 8034], osteopontin [RayBiotech, ELH-OPN-001], osteocalcin [TECOMedical, no. 8002], matrix γ-carboxyglutamic acid protein (MGP) [USCN Life Science, E91477Hu], fetuin A [TECOMedical, no. KT-800]), vascular remodelling (MMP-2, MMP-9, TIMP-1, TIMP-2 [Raybiotech: ELH-MMP2-001, ELH-MMP9-001, ELH-TIMP1-001, ELH-TIMP2-001]), cholesterol transport (ATP-binding cassette transporters ABCA1 [Uscn Life Science Inc., E91242Hu], ABCG1 [Uscn Life Science Inc., E91288Hu]) as well as renalase [USCN Life Science, E92845Hu] were measured in order to analyse their influence on cardiovascular risk in CKD patients. Levels of these proteins were determined by the ELISA method according to the manufacturer’s instructions.

Levels of biochemical markers such as total cholesterol, low-density lipoprotein (LDL), high-density lipoprotein (HDL), triglyceride (TG), albuminuria, serum calcium and phosphate, Fe, total iron-binding capacity (TIBC), C-reactive protein (CRP), alkaline phosphatase activity, creatinine, urea, uric acid, total protein, the level of fibrinogen and D-dimers were also determined. Estimated glomerular filtration rate (GFR-MDRD) was calculated using the Modification of Diet in Renal Disease:


In addition, cardiac echocardiography was performed to assess heart condition and function, the presence of left ventricular hypertrophy and systolic and diastolic function disturbances. The following cardiac indexes were measured: end-systolic interventricular septum thickness (IVSs), end-diastolic interventricular septum thickness (IVSd), left ventricle mass (LV mass), diameter of the left ventricle during systole (LVsys) and diastole (LVdias), left atrial (LA) size, right ventricle (RV) size, aorta, mitral insufficiency (IM), aortic insufficiency (IA), tricuspid insufficiency (IT), early (E) to late (A) ventricular filling velocities ratio (E/A), early transmitral velocity (E’), ratio of early transmitral velocity to mitral annular early diastolic velocity (E/E’), deceleration time (DT), ejection fraction (EF%), valve calcification/fibrosis.

### Statistical analysis

Results were expressed as mean with standard deviation (mean ± SD) for continuous variables with normal distribution or as median with interquartile range (median, 25%-75%) in all other cases. The Shapiro-Wilk test was used to verify normal distribution of variables and Levene test to analyse the homogeneity of variance. Standard Student t test for the comparison of data showing no departures from normality, and the non-parametric Mann–Whitney U test for the remaining variables were used. For detailed multiple comparisons (more than two groups) one-way ANOVA with post hoc Tukey tests or non-parametric analysis of variance (Kruskal-Wallis test) with post hoc Conover-Inman tests was used, respectively. The χ^2^ test of independence was used for the analysis of discontinuous variables. Calculations were performed using Statistica^®^ 10 (StatSoft, Inc., Tulsa, OK, USA).

## Results

### The characteristics of studied groups

The average age of patients in the study group (all CKD stages) was 67.1 ± 12.9 years and 59.4 ± 10.0 in the control group. Men were in the majority in the study groups while women were in the majority in the control group. Diabetes, hypertension, atrial fibrillation, general atherosclerosis and CRP > normal level occurred much more often in the study group than in controls. eGFR-MDRD was also much higher in the control group compared to study group, which is not surprising. No significant differences in the prevalence of obesity and lipid disorders were observed between groups. Demographic data are summarized in Table 1.

**Table 1:** **Detailed characteristics of included patients**

	Control group N = 45	CKD all stages N = 132	p	Stage I/II N = 30	Stage III N = 30	Stage IV N = 30	Stage V N = 49	p
**Age**	59.4 ± 10.0	67.1 ± 12.9	**P < 0.0001**	69.0 ± 10.7^1^	64.5 ± 14.8	67.9 ± 14.4	67.3 ± 12.1^1^	**P = 0.005**
**Sex, male (%)**	35.6%	56.1%	**P = 0.017**	51.9%	51.6%	36.0%	(71.4)^3^	**P = 0.0053**
**DM (%)**	11.1%	34.1%	**P = 0.003**	22.2%	32.3^1^	24.0%	46.9^3^	**P = 0.003**
**Hypertension (%)**	48.9%	88.6%	**P < 0.0001**	92.6%^3^	90.3%^3^	92.0%^3^	83.7%^3^	**P < 0.0001**
**Lipid disorders (%)**	37.8%	31.1%	NS	25.9%	38.7%	40.0%	24.5%	NS
**Obesity (%)**	33.3%	37.1%	NS	51.9%	45.2%	32.0%	26.5%	NS
**General atherosclerosis (%)**	6.7%	25.0%	**P = 0.008**	25.9%	16.1%	24.0%	30.6%^2^	**P = 0.05**
**K** ^**+**^ **[mmol/l]**	4.27 ± 0.52	4.64 ± 0.73	**P = 0.007**	4.59 ± 0.56	4.31 ± 0.48	4.62 ± 0.58	4.9 ± 0.9^2,7^	**P = 0.003**
**Haemoglobin [g/dl]**	13.4 ± 1.5	11.3 ± 2.0	**P < 0.0001**	11.9 ± 1.8^1^	12.6 ± 1.8	11.2 ± 2.0^3^	10.2 ± 1.6^3,5,9^	**P < 0.0001**
Platelet [*10 ^**3**^ **/μl]**	246.3 ± 70.6	244.1 ± 95.3	NS	250.4 ± 108.6	243.2 ± 71.4	250.0 ± 120.2	238.0 ± 89.1	NS
Monocytes [*10 ^**3**^ **/μl]**	0.44 ± 0.14	0.49 ± 0.23	NS	0.49 ± 0.21	0.50 ± 0.25	0.57 ± 0.29	0.46 ± 0.19	NS
**CRP [mg/l]**	1.7 (0.8-4.8)	6.4 (2.3-12.4)	**P < 0.0001**	6.4 (2.0-12.7)^1^	3.9 (1.9-7.9)	7.6 (2.4-18.1)^2^	7.9 (3.4-17.1)^3^	**P < 0.0001**
**ALP [U/l]**	82.2 ± 34.9	94.5 ± 55.4	NS	93.3 ± 32.8	69.2 ± 31.7	111.8 ± 101.9	100.8 ± 40.1^8^	**P = 0.004**
	79.5 (59.2-97.0)	86.0 (66.0-108.2)		88.5 (66.7-106.6)	67.0 (51.0-82.0)	82.0 (66.0-107.0)	97.0 (73.5-117.0)	
**APTT [s]**	29.4 ± 3.1	37.4 ± 11.4	**P < 0.0001**	34.2 ± 5.1^1^	35.5 ± 12.4	40.0 ± 14.8^2^	38.2 ± 10.4^3^	**P < 0.0001**
**APTT ratio**	0.95 ± 0.21	1.13 ± 0.37	**P = 0.002**	1.06 ± 0.19	1.10 ± 0.45	1.16 ± 0.43	1.14 ± 0.34^2^	**P = 0.019**
**Fe [μmol/l]**	16.9 ± 6.0	11.5 ± 6.2	**P < 0.0001**	10.5 ± 5.7^2^	15.3 ± 8.1	11.0 ± 4.6^2^	10.4 ± 5.5^3,7^	**P < 0.0001**
**TIBC [μmol/l]**	49.0 (45.0-50.0)	44.6 (36.5-56.8)	NS	54.2 (35.7-61.0)	61.0 (52.5-145.5)	45.0 (36.5-57.1)^7^	39.9 (34.7-47.6)^3^	**P < 0.0001**
**Ferritin [ng/ml]**	271.0 (8.5-328.0)	228 (105.5-680.5)	NS	182.5 (66.0-421.2)	134.9 (49.0-221.0)	277 (153.3-868.0)	444 (137.0-844.0)	NS (P = 0.097)
**Ca** ^**2+**^ **[mmol/l]**	2.36 ± 0.11	2.24 ± 0.23	**P = 0.006**	2.25 ± 0.20	2.34 ± 0.20	2.30 ± 0.21	2.15 ± 0.24^3,9,10^	**P < 0.0001**
**Inorganic P [mmol/l]**	1.12 ± 0.15	1.49 ± 0.49	**P < 0.0001**	1.49 ± 0.66	1.23 ± 0.24	1.39 ± 0.36^1^	1.67 ± 0.48^3,8^	**P < 0.0001**
**LDL [mmol/l]**	3.50 ± 0.76	3.08 ± 1.66	**P = 0.001**	2.96 ± 1.08	3.25 ± 1.68	3.86 ± 2.77	2.69 ± 1.01^2^	**P = 0.007**
**HDL [mmol/l]**	1.34 ± 0.36	1.26 ± 0.44	NS	1.37 ± 0.42	1.21 ± 0.38	1.38 ± 0.58	1.16 ± 0.39	NS (P = 0.077)
**TG [mmol/l]**	1.6 ± 1.0	1.91 ± 1.00	NS (0.063)	1.7 ± 0.7	2.3 ± 1.3	1.8 ± 1.0	1.8 ± 0.9	NS
**Total cholesterol [mmol/l]**	5.35 ± 0.79	5.13 ± 2.12	**P = 0.033**	5.05 ± 1.14	5.29 ± 1.72	6.08 ± 3.81	@4.54 ± 1.20	**P = 0.035**
**Albumin [g/l]**	44.8 ± 4.6	38.6 ± 6.1	**P < 0.0001**	38.1 ± 6.6^1^	39.5 ± 6.8	39.4 ± 6.1	37.9 ± 5.5^3^	**P = 0.002**
**Urea [mmol/l]**	5.7 ± 2.0	17.1 ± 10.3	**P < 0.0001**	14.1 ± 8.5^3^	13.0 ± 12.8^2^	17.5 ± 7.2^3^	21.2 ± 9.6^3,9^	**P < 0.0001**
	5.1 (4.5-6.4)	15.5 (9.1-22.5)		11.5 (7.0-24.2)	9.8 (6.7-13.5)	17.0 (11.7-21.7)	20.6 (15.8-25.3)	
**Creatinine [μmol/l]**	83.1 ± 14.5	330.1 ± 234.9	**P < 0.0001**	290.6 ± 247.7^3^	159.6 ± 81.8^3^	260.2 ± 139.5^3^	502.5 ± 228.2^5,9^	**P < 0.0001**
	82.0 (75.0-92.0)	264 (131.7-470.5)		227 (109–415)	137 (113–174)	232 (172.5-300)	497 (329–677)^3^	
**Total protein [g/l]**	69.5 ± 3.9	66.8 ± 10.0	NS	69.2 ± 10.5	63.7 ± 9.9	70.7 ± 9.8	65.4 ± 9.2	NS (P = 0.084)
**Uric acid [μmol/l]**	305.0 ± 83.9	416.2 ± 128.3	**P < 0.0001**	442.8 ± 122.8^3^	418.3 ± 117.7^2^	459.8 ± 134.7^3^	375.7 ± 126.5	**P < 0.0001**
**eGFR MDRD**	74.0 (65.2-83.5)	21 (12.2-42.0)	**P < 0.0001**	25.0 (14.7-53.2)^3^	42.0 (28.0-55.0)^1^	21.4 (14.9-29.0)^3^	12.0 (8.0-16.0)^3,4,9^	**P < 0.0001**
**IVsd [mm]**	13.6 ± 1.9	15.0 ± 2.6	**P = 0.009**	14.1 ± 1.8	14.7 ± 2.5	14.1 ± 2.1	16.3 ± 2.9^3,4,10^	**P < 0.0001**
**IVss [mm]**	15.1 ± 1.4	15.5 ± 2.1	NS	16.0 ± 2.1	15.4 ± 1.9	15.0 ± 2.2	15.5 ± 2.2	NS
**LV mass [g]**	234.4 ± 51.1	345.0 ± 126.8	**P < 0.0001**	293.2 ± 72.5	326.8 ± 117.5	310.1 ± 96.0	403.5 ± 148.7^3,5,10^	**P < 0.0001**
**LV hypertrophy [%]**	95.2(%)	94.2%	NS	92.3%	96.6%	90.0%	95.6%	NS
**LV sys [mm]**	37.1 ± 4.1	40.7 ± 7.6	**P = 0.045**	38.5 ± 5.7	39.9 ± 5.7	38.7 ± 6.3	43.2 ± 9.5^1^	**P = 0.035**
**LV dias [mm]**	44.1 ± 7.0	48.7 ± 7.2	**P = 0.007**	46.0 ± 5.0	47.4 ± 6.4	47.7 ± 5.7	51.5 ± 8.5^2^	**P = 0.003**
**LA [mm]**	40.6 ± 2.9	42.4 ± 4.1	**P = 0.036**	41.3 ± 2.9	41.2 ± 3.9	41.7 ± 3.2	44.2 ± 4.6^2,4,8^	**P < 0.0001**
**RV [mm]**	26.0 ± 3.3	27.5 ± 3.8	NS	27.6 ± 2.1	26.8 ± 4.7	26.9 ± 3.8	28.1 ± 3.8	NS
**E/A**	0.87 ± 0.27	1.09 ± 0.59	NS	1.09 ± 0.73	0.80 ± 0.17	1.14 ± 0.18^7^	1.21 ± 0.66	**P = 0.014**
**E/E’**	7.9 ± 1.7	10.7 ± 4.8	NS	8.6 ± 4.2	8.8 ± 4.1	12.6 ± 4.6	12.3 ± 4.9	NS
**Dec (DT)**	209.6 ± 56.2	270.6 ± 123.6	NS	301.5 ± 157.9	321.3 ± 132.8	213.2 ± 73.4	246.6 ± 98.2	NS
**Contractility disorders [%]**	52.4%	62.2%	NS	36.8%	45.0%	43.8%	88.4%^2,6,9,11^	**P = 0.0001**
**EF%**	53.0 ± 3.5	48.5 ± 8.6	**P = 0.012**	50.8 ± 4.5	51.1 ± 8.4	49.9 ± 10.2	45.5 ± 8.9^7^	**P = 0.001**
**Mitral valve fibrosis [%]**	63.6%	82.4%	NS (P = 0.079)	87.0%	73.9%	70.6%	88.9%	NS
**Aortic valve fibrosis [%]**	13.6%	12.0%	NS	8.7%	8.7%	11.8%	15.6%	NS
**Mitral valve calcification [%]**	9.1%	21.3%	NS	8.7%	13.0%	11.8%	15.6%	NS
**Aortic valve calcification [%]**	9.5%	39.8%	**P = 0.008**	26.1%	21.7%	23.5%	35.6%	NS
**IM [%]**	63.6%	71.6%	NS	65.2%	54.2%	64.7%	86.7%^2^	**P = 0.045**
**IA [%]**	4.8%	12.8%	NS	13.0%	8.3%	5.9%	17.8%	NS
**IT [%]**	36.4%	58.3%	NS (P = 0.059)	47.8%	43.5%	47.1%	75.6%^2,4,7^	**P = 0.012**
**Fetuin A [ng/ml]**	37.5 (31.6-126.6)	33.2 (27.5-89.4)	**P = 0.018**	70.6 (30.9-119.7)	34.1 (28.5-92.5)	31.4 (26.2-114.0)	29.8 (25.5-37.5)^2,4^	**P = 0.002**
**Osteocalcin [ng/ml]**	9.1 ± 6.0	13.6 ± 10.3	**P = 0.05**	10.6 ± 9.0	8.4 ± 5.8	11.7 ± 9.8	19.2 ± 11.1^3,4,9^	**P < 0.0001**
	8.2 (5.1-13.0)	10.5 (5.0-23.1)		6.7 (4.3-19.1)	7.6 (4.3-11.9)	9.2 (4.5-17.9)	22.9 (8.5-28.2)	
**Osteopontin [ng/ml]**	8.8 (7.2-37.8)	9.3 (7.8-17.8)	NS	9.4 (6.7-20.9)	8.1 (7.4-16.1)	9.8 (8.1-25.2)	9.3 (8.0-13.8)	NS
**Osteoprotegerin [pmol/l]**	7.6 ± 5.5	8.5 ± 6.2	NS	7.5 ± 6.9	5.8 ± 4.6	8.2 ± 6.0	10.8 ± 6.2^2^	**P = 0.005**
	5.7 (2.9-12.0)	7.4 (3.5-12.2)		7.2 (1.1-11.0)	5.2 (1.1-9.5)	6.3 (3.7-11.6)	10.1 (6.1-14.4)	
**MGP [ng/ml]**	92.9 ± 28.5	86.7 ± 36.7	NS	77.1 ± 43.5	83.1 ± 33.9	89.5 ± 32.1	92.6 ± 36.7	NS
**ABCG1 [ng/ml]**	2.25 (0.98-2.58)	1.93 (0.72-2.43)	NS	1.94 (1.64-2.34)	1.86 (0.59-2.36)	2.19 (0.89-2.63)	1.84 (0.46-2.49)	NS
**ABCA1 [ng/ml]**	0.70 (0.62-0.75)	0.60 (0.44-0.74)	NS (P = 0.079)	0.71 (0.55-0.88)	0.51 (0.41-0.71)	0.59 (0.42-1.02)	0.58 (0.30-0.71)	NS (P = 0.054)
**MMP-2 [ng/ml]**	94.8 (54.2-166.3)	129.9 (73.4-219.3)	**P = 0.045**	206.9 ± 142.7	202.5 ± 152.3	160.6 ± 130.0	130.7 ± 105.8	**P = 0.008**
	113.1 ± 75.0	166.0 ± 129.9		166.9 (86.4-304.5)^1^	156.1 (105.7-274.2)^1^	136.5 (70.6-225.1)	103.4 (63.4-160.1)	
**MMP-9 [ng/ml]**	21.5 ± 6.4	22.5 ± 7.1	NS	20.1 ± 7.4	21.9 ± 6.2	22.7 ± 8.0	23.9 ± 6.9	NS
**TIMP-1 [ng/ml]**	21.8 ± 3.7	21.4 ± 2.4	NS	19.8 ± 3.5	21.8 ± 2.2	21.7 ± 1.9	21.9 ± 1.5^4^	**P = 0.048**
**TIMP-2 [ng/ml]**	22.1 ± 5.1	25.4 ± 7.0	**P = 0.005**	23.5 ± 6.4	23.9 ± 5.5	25.3 ± 6.5	27.3 ± 8.0^2^	**P = 0.003**
**MMP-2/TIMP-2**	4.5 (2.5-7.9)	6.1 (3.0-11.4)	NS (P = 0.056)	11.0 (5.7-17.9)^2^	7.4 (4.4-14.7)	6.3 (3.3-11.4)	3.8 (2.0-8.4)^5^	**P = 0.001**
**MMP-9/TIMP-1**	1.09 (0.65-1.28)	1.15 (0.75-1.37)	NS	1.16 ± 0.56	1.17 ± 0.71	1.08 ± 0.41	1.11 ± 0.37	NS
**Renalase [ng/ml]**	251.0 ± 157.0	316.1 ± 155.3	**P = 0.026**	354.5 ± 181.6	235.3 ± 152.3	279.4 ± 162.2	369.0 ± 110.5^2,5^	**P < 0.0001**

^1^vs control, p < 0.05; ^2^vs control, p < 0.01; ^3^vs control, p < 0.001.

^4^vs stage I-II, p < 0.05; ^5^vs stage I-II, p < 0.01; ^6^vs stage I-II, P < 0.001.

^7^vs stage III, p < 0.05; ^8^vs stage III, p < 0.01; ^9^vs stage III, p < 0.001.

^10^vs stage IV, p < 0.05; ^11^vs stage IV, p < 0.001.

### The analysis of biochemical parameters

The analysis of biochemical parameters and echocardiographic indices in study group and controls was also performed (results are summarized in Table 1). Statistically significant differences in the level of K^+^, haemoglobin, APTT, Fe, LDL, total cholesterol, inorganic phosphate and calcium ions between control and study group were observed. These differences were also noticeable between patients with various CKD stages. Moreover, in CKD patients markers of kidney damage (urea, creatinine, albumin and uric acid) were significantly higher than in controls.

### The analysis of echocardiographic indices

Echocardiographic examination revealed that patients with CKD in comparison with the control group had significantly greater interventricular septal end diastolic dimension, higher left ventricular mass and much larger systolic and diastolic dimensions of the left ventricle, indicating pathological cardiac hypertrophy. These patients also had a significantly greater left atrial dimension. Chronic kidney disease patients were also more likely to have aortic valve calcification and their ejection fraction was significantly lower than in the control group. These observations confirm the adverse effect of renal disease on the cardiovascular system. Although diastolic dysfunction is frequent in patients with CKD, there were no significant differences in its indices, such as E/A and E/E’ between study and control group.

### The analysis of proteins concentrations

Analysis of the concentrations of proteins associated with bone metabolism revealed no statistically significant differences in the level of osteoprotegerin, osteopontin, or MPG between the control group and patients with chronic kidney disease (all stages). However, it was observed that the concentration of fetuin A in study group was significantly lower than in controls (33.2 vs. 37.5 ng/ml, p = 0.018) while the level of osteocalcin was significantly higher in patients with CKD (13.6 ± 10.3 vs. 9.1 ± 6.0 ng/ml, p = 0.05). Moreover, differences in fetuin A, osteocalcin and osteoprotegerin between groups of patients in various CKD stages were seen. Fetuin A level was highest in patients with mild kidney disease and lowest in dialysis patients (p = 0.002); osteocalcin and osteoprotegerin concentrations were highest in dialysis patients and lowest in stages I/II (p < 0.0001 and p = 0.005, respectively). The results are summarized in Table 1. The study of matrix metalloproteinases and their inhibitors revealed that the level of MMP-2 was much higher in patients with chronic kidney disease in comparison to the control group (p = 0.045) and that statistically significant differences between various stages of CKD were also present (p = 0.008). Its highest values were seen in patients with mild kidney disease (stage I/II) then it gradually decreased to reach the lowest value in patients with stage V/dialysis. In case of MMP-9 level only a slight tendency to increase with the worsening of kidney function was noted. Significant differences in proteins concentration between study and control groups were also observed in case of metalloproteinase inhibitor TIMP-2 (p = 0.005) and in the level of TIMP-1 and TIMP-2 between various CKD stages (p = 0.048 and p = 0.003, respectively). The highest concentrations of TIMP-1 and TIMP-2 were observed in end-stage renal disease patients and the lowest in I/II CKD patients. Furthermore, it was noted that the ratio MMP-2/TIMP-2 was significantly lower in V/dialysis patients than in patients with mild renal impairment (p = 0.001). The level of renalase also differed significantly between CKD patients and control group (p = 0.026) and between patients with various CKD stages (p < 0.0001).

### The analysis of relationship between protein concentration and biochemical parameters

The analysis of the relationship between the incidence of both calcification and fibrosis and sex (male), age, level of calcium ions and inorganic phosphate in plasma demonstrated that the risk of mitral valve fibrosis is increased every year 1.15-fold, the risk of aortic valve fibrosis 1.02-fold and the risk of mitral valve calcification by 1.05, regardless of the concentration of Ca^2+^, inorganic phosphate level and sex. The results are shown in Table 2.

**Table 2:** **Correlation between the presence of fibrosis and calcification and selected parameters**

	Sex (male)	Age	Ca ^2+^	Inorganic phosphate
Mitral valve fibrosis	1.39 (0.36-5.28) NS	1.15 (1.07-1.24) P < 0.0001	0.98 (0.06-16.94) NS	3.1 (0.56-17.19) NS
Aortic valve fibrosis	0.33 (0.09-1.22) NS	1.02 (0.97-1.08) P < 0.0001	0.53 (0.04-7.51) NS	1.13 (0.36-3.47) NS
Mitral valve calcification	1.64 (0.59-4.51) NS	1.05 (1.00-1.09) P = 0.037	0.25 (0.02-2.41) NS	0.84 (0.30-2.34) NS
Aortic valve calcification	1.61 (0.65-3.97) NS	1.04 (1.00-1.08) P = 0.054	0.86 (0.11-6.82) NS	4.37 (1.57-12.15) P = 0.005

An analysis of the relationship between concentrations of studied proteins and biochemical parameters was also performed. In the present study an association between serum osteocalcin, osteoprotegerin and fetuin A and the level of inorganic phosphate and between osteoprotegerin levels and CRP was found. Moreover, there was a relationship between fetuin A concentration and the level of calcium ions. In the present study, no association was observed between the concentration of transport proteins ABCA1 and ABCG1 and HDL cholesterol, LDL, total cholesterol and triglycerides levels. There was also no relationship between the level of these proteins and a marker of inflammation (CRP) or a marker of renal injury (creatinine). A weak association was observed between the concentration of metalloproteinase-9 and the level of CRP (p = 0.066) and a stronger one between TIMP-2 and MDRD-GFR (p = 0.007). The concentration of TIMP-2 also correlated with the level of iron (p = 0.018). In this study, a relationship between the concentration of renalase and the kidney injury markers creatinine and urea was noted.

Statistically significant results of the correlation analysis are presented in Table 3.

**Table 3:** **Results of analysis of correlation between concentration of selected proteins and biochemical parameters**

	Ca ^2+^	Inorganic P	CRP		HDL	LDL	TCH	TG	CRP	GFR-MDRD	Ca ^2+^	Inorganic P	Fe
Osteopontin	0.024 NS	0.009 NS	0.094 NS	ABCA1	0.012 NS	0.016 NS	0.127 NS	0.032 NS	-0.154 NS				
Osteoprotegerin	-0.117 NS	0.385 P < 0.0001	0.290 P = 0.001	ABCG1	0.150 NS	-0.109 NS	0.052 NS	-0.097 NS	-0.014 NS				
Osteokalcin	-0.144 NS	0.192 P = 0.041	0.019 NS	MMP-2	-0.079 NS	-0.001 NS	0.096 NS	0.125 NS	0.113 NS	0.048 NS	0.095 NS	-0.119 NS	0.149 NS
Fetuin A	0.270 P = 0.003	-0.296 P = 0.001	-0.117 NS	MMP-9	-0.075 NS	-0.060 NS	-0.104 NS	-0.040 NS	0.167 NS (p = 0.066)	-0.162 NS	-0.038 NS	0.026 NS	-0.067 NS
MGP	-0.41 NS	-0.005 NS	0.034 NS	TIMP-1	-0.062 NS	0.084NS	-0.067 NS	-0.093 NS	0.007 NS	-0.169 NS	-0.083 NS	0.105 NS	-0.095 NS
	Creatinine	Urea	CRP	TIMP-2	-0.019 NS	0.029 NS	0.050 NS	0.128 NS	-0.028 NS	-0.272 P = 0.007	-0.091NS	0.061 NS	-0.223 P = 0.018
Renalase	0.410	0.376	0.073 NS										
	P < 0.0001	P < 0.0001											

## Discussion

Biochemical and physiological abnormalities in chronic kidney disease are associated with the attempts of the human body to maintain homeostasis despite progressive loss of nephrons and impaired kidney function. Multiple adaptive and maladaptive processes occurring at that time affect mainly the cardiovascular, neurological, haematological, musculoskeletal, and immunological systems [13, 14].

This preliminary study analysed the possible markers of increased cardiovascular risk and calcification processes occurring in CKD patients.

The results of analysis of biochemical parameters – significantly higher levels of K^+^, lower haemoglobin and iron, as well as higher levels of CRP, inorganic P and triglycerides in patients with CKD stages I-V in comparison with the control group – are consistent with the results of other studies [15, 16] and are related to kidney damage. Decreased levels of haemoglobin are not surprising since CKD patients frequently suffer from anaemia. The main factors leading to the development of anaemia in this group of patients are blood loss, shortened red cell life span, vitamin deficiency, the ‘uremic milieu’, iron and erythropoietin (EPO) deficiency as well as reduced ability to bind Fe, inflammation and hyperparathyroidism [13]. The presence of the inflammatory state in CKD patients is confirmed by high CRP levels. Significantly higher concentrations of urea, creatinine and uric acid in patients with CKD stages I-V are not astonishing, since these are well-known markers of kidney damage. In CKD patients iron homeostasis is altered. The level of transferrin is decreased, which negatively affects the capacity of the iron-transporting system, and the release of iron stored in macrophages and hepatocytes is complicated [13, 17]. It is believed that lipid abnormalities are a common phenomenon in patients with kidney disease. In the early stages of kidney disease the lipid profile may remain in the normal range, but with the progression of CKD hypertriglyceridaemia, elevated concentrations of total cholesterol, VLDL (very low density lipoprotein), IDL (intermediate density lipoprotein), LDL and decreased HDL cholesterol appear [18]. In this study significantly lower concentrations of total cholesterol and LDL cholesterol in CKD patients compared to healthy volunteers were observed. Triglyceride concentrations are usually high in CKD patients and this disturbance is seen in early stages of CKD and is present in up to 70% of ESRD patients [18, 19]. High-density lipoprotein (HDL) cholesterol is subnormal in the majority of patients, which is consistent with the results of this study. Uraemia causes qualitative changes in the composition of LDL, increases the number of small, dense LDL particles and facilitates their oxidation and glycation. LDL cholesterol in CKD may be normal or lower; however, the proportion of small dense LDL particles, which are considered to be highly atherogenic, is increased [20]. According to studies [18], the profile of dyslipidaemia is similar to that found in patients with accelerated atherosclerosis. It seems that greater severity of atherosclerosis in patients with CKD may also be associated with disorders of calcium-phosphate metabolism. Impaired excretion of phosphate leads to its increased concentration, to binding with calcium in tissues and to the accumulation of such compound in the arteries, which increases the risk of heart attack and stroke [21]. High levels of phosphate usually appear when the GFR falls below 60 ml/min and worsens with the deterioration of renal excretory functions. Hyperphosphataemia stimulates parathyroid glands to increased secretion of parathyroid hormone (PTH), which in turn increases the release of phosphate from the bone, contributing to further accumulation of phosphate in serum. High levels of inorganic phosphate in CKD patients observed in this study are in accordance with the results of other studies.

Progressive left ventricular enlargement seems to be the most characteristic morphological pattern of dialysis patients and is one of the key prognostic factors for cardiovascular mortality rates in ESRD patients [22, 23]. In this study systolic and diastolic dimensions of the left ventricle as well as LV mass differed significantly between CKD patients and controls. These parameters were much higher in ESRD patients than in patients with CKD stage I/II According to Zoccali et al. [24], patients in the highest tertiles of change in LV mass undergoing haemodialysis had a 50% mortality risk and >85% CV event risk at 3 years. Moreover, another study [25] demonstrated that a 10% decrease in left ventricle (LV) mass (∼29 g) was associated in haemodialysis patients with a 28% decrease in mortality risk from cardiovascular causes over an almost 5-year follow-up. Numerous studies have demonstrated that diastolic dysfunction, which is common in chronic kidney disease (CKD), accounts for 40%-66% of cardiovascular complications [26]. The proper diagnosis of LV diastolic dysfunction is very important since it has been demonstrated that it provides independent, prognostic value for long-term mortality and cardiovascular death in patients with end-stage renal disease [27]. Also the study of Iwabuchi et al. [28] demonstrated that E/E’ ratio is a significant predictor of cardiovascular events in haemodialysis patients with preserved LV systolic function and is better than other echocardiographic parameters. However, in this study significant differences in indices of diastolic dysfunction (E/A and E/E’) between study group and controls were not observed. This may be due to the fact that the studied groups were not large.

Lower ejection fraction and frequent mitral and tricuspid regurgitation observed in CKD patients in this study is consistent with the results of other studies [29]. It has been observed that the calcification process is common and more severe in the CKD population compared with healthy people [30]. According to studies, in addition to arterial wall calcification in patients with CKD valvular calcification is frequently observed. These changes are localized mainly within the mitral valve ring and extend towards valve leaflets [31, 32]. It is believed that the severity of vascular and valvular calcification in patients undergoing haemodialysis correlates with the incidence of cardiovascular complications and is a predictor of cardiovascular mortality [31, 33]. In this study aortic and mitral valve calcification was observed, and they were much more frequent in CKD patients than in healthy volunteers. In the study of Leskinen et al. [34] combined prevalence of mitral or aortic valve calcification was 31% in pre-dialysis patients, 50% in dialysis patients and 12% in controls (p = 0.001). In the Multi-Ethnic Study of Atherosclerosis in patients with moderate CKD and no clinical cardiovascular disease, calcification of the aortic valve and mitral valve was present in 25 and 20% of people respectively [35]. It is believed that the severity of vascular and valvular calcification in haemodialysis patients correlates with the incidence of cardiovascular complications and is a predictor of cardiovascular mortality [32, 36]. The contribution of traditional risk factors does not fully explain the high frequency of cardiovascular disease, and thus scientists look for new mechanisms involved in its pathogenesis [37–42]. The coronary artery calcification score (CACS) may be as much as 5-fold higher in patients on maintenance haemodialysis than in age-matched non-CKD patients [43]. Nowadays it seems that the differentiation of vascular smooth muscle cells (VSMCs) into chondrocytes or osteoblast-like cells is a key element in vascular/valvular pathogenesis [44]. Many bone-associated proteins, including osteocalcin (OC), matrix γ-carboxyglutamic acid protein (MGP), osteoprotegerin (OPG), osteopontin (OPN) and fetuin A, are expressed in atherosclerotic plaques and participate in atherosclerotic calcification. In an *in vitro* study, exogenous osteocalcin inhibited the process of calcification [45]. According to the literature, its concentration in patients with CKD is high. Increased serum concentration of osteocalcin in patients with CKD may be associated with reduced renal clearance, increased bone metabolism or a combination of both phenomena [46, 47]. In this study, only concentrations of fetuin A (associated with the presence of massive calcifications of soft tissue and widespread arterial calcification [48, 49]) and osteocalcin (which is considered to be the key factor in the development of atherosclerosis) significantly differed between patients with chronic kidney disease and controls. The concentration of osteocalcin was lowest in patients with stage I/II CKD and gradually increased to reach its highest value in patients with stage V/dialysis. According to Delmas et al. [47], raised levels of circulating osteocalcin in patients with mild or moderate renal impairment reflect enhanced bone metabolism rather than decreased renal filtration. In this study, fetuin A level decreased significantly with the progression of CKD, which is consistent with the results of other studies [50, 51]. Schafer et al. [52] suggested that fetuin A deficiency in haemodialysis patients may be responsible for the development of calciphylaxis, which includes changes in small arterioles. A low level of fetuin A in these patients is considered an independent risk factor for increased mortality from cardiovascular causes [53]. Furthermore, Wang et al. [54] demonstrated that serum concentration of fetuin A decreased as MIA syndrome intensified. However, our study failed to observe such an association, probably due to the quite small study group. Also, osteoprotegerin deficiency leads to vascular calcification since it inhibits osteoclast differentiation and is a crucial modulator of bone resorption [37, 55]. In this study, the concentration of osteoprotegerin in controls and study groups did not differ significantly; however, the authors observed its lowest values in subjects with I/II stage CKD and highest in patients with stage V/dialysis. The results showing an increase of osteoprotegerin levels with increasing renal impairment are consistent with the results of other studies. The study by Amann K [56] showed that levels of osteoprotegerin in CKD patients are elevated, which is a defence mechanism against vascular calcification. Bucay et al. [48] demonstrated that mice lacking the osteoprotegerin gene had severe osteoporosis accompanied by rapid progression of calcification of arteries, while the exogenous administration of OPG inhibited the vascular calcification process. On the other hand, it was found that the expression of OPG in the atheromatous plaque and in vascular smooth muscle cells is very high and it is regulated by a number of inflammatory cytokines [57]. Other studies have shown that both the process of vascular calcification [58] and the aortic calcification index [59] correlated with serum concentration of OPG in patients on haemodialysis. It seems that increased levels of OPG in patients with CKD can affect both the presence of systemic inflammation and local dysfunction of vascular endothelium [60]. In this study, there are slight differences in osteopontin concentration between groups, however, they did not reach statistical significance level, probably due to small study groups.

Because the calcification process is in part associated with the degradation of elastin, this study examined the level of metalloproteinases (MMPs) and their inhibitors. Degradation of elastin by matrix metalloproteinases (MMP-2 and -9) [37, 61, 62] activates the mitogen-activated protein kinase (MAPK) signalling pathway and may further result in the induction of Cbfa1/Runx2 and sequentially initiate the transformation of VSMCs into osteoblast-like cells [37, 63]. Analysis of results of this study revealed significantly higher levels of MMP-2 in patients with chronic kidney disease in comparison to the control group. Also the study of Musial et al. [33] demonstrated that concentrations of MMP-2 increased with the decline of renal function. Similarly, Nagano et al. [64] observed a correlation between serum concentration of MMP-2 and kidney function parameters and confirmed that MMP-2 can be an indicator of the severity of atherosclerosis in CKD patients. Apart from MMP-2, the role of MMP-9 in the pathogenesis of vascular changes in atherosclerosis has also been established. The study by Chen et al. [65] revealed increased expression of MMP-2 and MMP-9 in areas of aorta calcification. Moreover, MMP inhibitors decreased calcification of aorta rings from normal and CKD rats, which may suggest that degradation of the extracellular matrix is a critical step in the pathogenesis of arterial calcification in CKD [65]. In this study, significant differences in concentration of TIMP-2 between CKD patients and controls and in metalloproteinase inhibitors TIMP-1 and TIMP-2 and the MMP-2/TIMP-2 ratio between CKD subjects in various stages were observed. The study of Musial et al. [33] demonstrated that concentrations of TIMP-1 and -2 start to increase in the advanced stages of CKD, so they are probably a response to the increased activity of matrix metalloproteinases.

A key stage in the atherosclerotic process is the formation of foam cells from macrophages. Numerous studies indicate that combined action of transport proteins ABCA1 and ABCG1 in macrophages are very important for the anti-atherogenic effect of HDL. ABCA1 plays an important role in reverse cholesterol transport; its activity is associated with HDL cholesterol concentration and atherosclerosis. ABCG1 enables transport of phospholipids and cholesterol from cell membranes to ApoAI-rich HDL precursors and can act synergistically with ABCA1 to remove cholesterol from cells [66]. Both transporters mediate macrophage cholesterol efflux, and protect against the formation of foam cells from macrophages, infiltration of inflammatory cells, apoptosis and atherosclerosis [67]. In humans, a homozygous variant of ABCA1 present in Tangier disease is associated with a very low level of HDL cholesterol, a high concentration of triglycerides, and six times higher risk of cardiovascular disease [68]. In turn, the disruption of the ABCG1 gene in mice fed with a high-fat diet results in a massive accumulation of lipid in macrophages in various tissues [69]. However, no significant differences in ABCA1 and ABCG1 between study and control groups were found. Increased levels of renalase in CKD patients, observed in this study, are consistent with the study of Desir et al. [70, 71]. Concentration of this protein was higher in patients with early stages of CKD than in the control group. Similar observations were made in the Malyszko et al. [72, 73] study, in which the renalase level was much higher in patients who had undergone renal transplant than in healthy subjects. Renalase concentration in patients with CKD correlates with increased plasma norepinephrine levels, systolic blood pressure and proteinuria, suggesting that this protein plays an important role in the metabolism of catecholamines, participates in the development of hypertension and cardiovascular disorders, and may directly or indirectly contribute to kidney damage [74] and increased mortality of CKD patients [75, 76].

Our study also revealed a correlation between osteocalcin level and inorganic P and between osteoprotegerin and inorganic P and CRP. In patients with CKD, it is well established that hyperphosphataemia is associated with the development of vascular calcification [38, 44, 77–82]. Recent studies have demonstrated that high extracellular phosphate levels induce the transformation of VSMCs into osteoblast-like cells, which suggests that vascular calcification is an active process. Moreover, elevated extracellular phosphate levels are associated with the increase in bone-associated proteins such as osteocalcin and osteopontin [38, 78]. Fetuin A correlated well with the level of calcium ions. Also in the study by Cagli et al. there was a positive correlation between serum fetuin A and calcium [80]. In this study, a relationship between the concentration of renalase and the kidney injury markers creatinine and urea was noted. In the study by Malyszko et al. [72], renalase levels in patients after kidney transplantation also correlated with plasma creatinine concentration and MDRD eGFR values.

### Limitations

Our study has some limitations. The number of participants included to the study is relatively small (139 patients with CKD and 45 healthy volunteers). Study and control groups differ in age due to the fact that it is difficult to find healthy people aged 60–70 years to match study group. There are also differences in other demographic data such as sex, diabetes mellitus and hypertension between groups.

## Conclusions

In conclusion, we showed that significantly decreased levels of fetuin A and increased concentration of osteocalcin were present in all patients with chronic kidney disease. Moreover, MMP-2 and TIMP-2 concentrations were much higher in patients with chronic kidney disease, suggesting that these factors may be involved in the pathogenesis of CAD in patients with CKD. Lack of significant differences in parameters associated with the transport of cholesterol may mean that commonly measured cholesterol level does not play the important role in the CV risk stratification in CKD patients. Moreover, according to literature, in uremic conditions HDL particles may lose their protective properties and also become atherogenic. The level of renalase was significantly higher in patients with CKD than in controls and it increased with the deterioration of renal function. The results suggest that that valvular calcifications may constitute a marker for atherosclerosis.
